# Factors Affecting Growth of Tengmalm’s Owl (*Aegolius funereus*) Nestlings: Prey Abundance, Sex and Hatching Order

**DOI:** 10.1371/journal.pone.0138177

**Published:** 2015-10-07

**Authors:** Markéta Zárybnická, Jan Riegert, Lucie Brejšková, Jiří Šindelář, Marek Kouba, Jan Hanel, Alena Popelková, Petra Menclová, Václav Tomášek, Karel Šťastný

**Affiliations:** 1 Department of Ecology, Faculty of Environmental Sciences, Czech University of Life Sciences Prague, Prague, Czech Republic; 2 Department of Zoology, Faculty of Science, University of South Bohemia in České Budějovice, České Budějovice, Czech Republic; University of Missouri Kansas City, UNITED STATES

## Abstract

In altricial birds, energy supply during growth is a major predictor of the physical condition and survival prospects of fledglings. A number of experimental studies have shown that nestling body mass and wing length can vary with particular extrinsic factors, but between-year observational data on this topic are scarce. Based on a seven-year observational study in a central European Tengmalm’s owl population we examine the effect of year, brood size, hatching order, and sex on nestling body mass and wing length, as well as the effect of prey abundance on parameters of growth curve. We found that nestling body mass varied among years, and parameters of growth curve, i.e. growth rate and inflection point in particular, increased with increasing abundance of the owl’s main prey (*Apodemus* mice, *Microtus* voles), and pooled prey abundance (*Apodemus* mice, *Microtus* voles, and *Sorex* shrews). Furthermore, nestling body mass varied with hatching order and between sexes being larger for females and for the first-hatched brood mates. Brood size had no effect on nestling body mass. Simultaneously, we found no effect of year, brood size, hatching order, or sex on the wing length of nestlings. Our findings suggest that in this temperate owl population, nestling body mass is more sensitive to prey abundance than is wing length. The latter is probably more limited by the physiology of the species.

## Introduction

In altricial birds, phenotypic characteristics of nestlings such as body mass and wing length can affect sibling competition [1,2], nestlings’ survival [3,4] and at which age each individual fledges [5]. During the post-fledgling period, the body characteristics affect fledgling survival, behavioural performance and recruitment into the breeding population [6–8]. Energy supply, which is determined by natural food availability and parental effort [9,10], is the main factor determining individual fledglings’ body mass [3,4,11–13] and enhances feather growth [13–15]. In species where asynchronous hatching seems to be an adaptation to unpredictable changes in food supply, the youngest nestling usually dies first due to its poor body condition (i.e. brood reduction theory; [16–20]). This effect is more pronounced during food scarcity [16,19,21]. Simultaneously, nestlings from enlarged broods usually reach a lower body mass and shorter wings compared to those from smaller broods, due to increased sibling competition [22–25]. Moreover, the larger sex usually grows faster than the smaller sex and reaches a higher body mass [26–29]. However, across bird species, particular factors can play different roles. For example, the body condition of Montagu’s harrier *Circus pygargus* fledglings does not vary according to sex and food abundance [30], the larger sex of marsh harrier *Circus aeruginosus* nestlings has no reduction effect on the smaller sex [28], and brood size in common grackles *Quiscalus quiscula* has no effect on nestling body mass [31]. Moreover, despite the reasonable amount of existing experimental studies on the differential quantity and quality of fledglings, observational studies remain scarce.

The Tengmalm’s owl *Aegolius funereus* is a nocturnal avian predator with a wide Holartic breeding range [32], feeding mainly on voles in northern areas, and voles and mice in temperate areas [33–36]. As in other raptors and owls, eggs are laid at an interval of 1–2 days, and incubation begins after the first or second egg has been laid. Consequently, the nestlings hatch asynchronously and the youngest nestlings usually suffer from a higher mortality risk due to a combination of starvation and sibling aggression [32,37]. The nestlings stay on the nest usually 32 days (range 27 ˗ 38 days) from hatching [5]. In Tengmalm’s owl, reversed size dimorphism reaches approximately 30–40% in terms of body mass during the breeding season due to female body reserves for egg production and successful incubation, although the difference is only 4% in body mass and 2.5% in wing length outside the breeding season [38,39]. Hipkiss et al. [3] found that female nestlings in northern populations attained 5% higher asymptotic mass than males, and that experimentally fed nestlings were heavier than control nestlings, suggesting that the females’ larger size gave them a competitive advantage against their male siblings during food scarcity. On the other hand, Kouba et al. [5] demonstrated that wing length is important measurement of individual quality in Tengmalm’s owl young because it determines the duration of the nestling period (i.e., duration of stay of individual nestlings at the nest box from hatching to fledging). However, between-year observational data on this topic are scarce, as well as no long-term study has focused on the effect of extrinsic factors such as prey abundance, brood size, hatching order, and sex on body mass and wing length in nestlings of raptors in general and Tengmalm’s owl in particular.

In this study, we analyse a seven-year observational data on nestling conditions from a central European population of the Tengmalm’s owl. Specifically, we examine the effect of year, brood size, hatching order, and sex on nestling body mass and wing length, as well as the effect of prey abundance on parameters of growth curve. We hypothesise that (i) body mass and wing length of nestlings (controlled for age) vary among years due to between-year variation in prey abundance, and growth parameters (i.e. asymptote, inflection point, and growth rate) increase with prey abundance, (ii) nestlings in smaller broods, as well as (iii) early-hatched brood-mates, attain a higher body mass and wing length (both controlled for age) than nestlings in larger broods and later-hatched brood-mates, and (iv) females attain a higher body mass than males, but not longer wings. To support our results, we also compare main growth parameters (asymptote, inflection point and growth rate) among years, hatching order groups and sexes.

## Materials and Methods

### Ethics statement

The project (evaluating small mammal abundances and handling with Tengmalm’s owl chicks) was approved by the Ministry of the Environment of the Czech Republic (permit No. 35016/02-OOP/8751/02, 530/758 R/08-Abt/UL, 01220/LP/2008, and 48429/ENV/14-2831/630/14). Owl chicks were handled and ringed under the Ringing Centre of the National Museum in Prague permit No. 329 and 942. Maximum effort was made to reduce handling time.

### Study area

The study was conducted in the northern part of the Czech Republic (50°N, 13°E), on the Ore Mountain plateau (elevation: 730–960 m a. s. l.; area: ca 100 km^2^), from 2006 to 2014, except 2007 (blood of nestlings not available) and 2013 (no nestlings hatched). The study site is characterized by a mosaic of small patches of mature Norway Spruce *Picea abies* forests, fragments of young secondary forests, and open areas with no trees; such a pattern resulted from significant impacts of air pollution towards the end of the 20th century (for details see [40]). In this study site, Tengmalm’s owls breed mainly in nest boxes (> 90% of breeding pairs), as naturally occurring cavities for nesting are scarce [40]. The nest boxes were evenly distributed within the study area, and their numbers varied between 116 and 212 (mean ± SE; 159.3 ± 11.5) in different years.

### Field procedures

Each year, all nest boxes were inspected from the onset of the breeding period (late March) to the end of the breeding period (July) in intervals of one to three weeks to detect new breeders. Nest boxes found to be occupied were subsequently revisited at a sufficient frequency to assess the number of eggs, hatchlings and hatching date (± 1 day). During the study period of 2006–2014 (except 2007 and 2013) we found a total of 116 nesting attempts from which 301 nestlings hatched. We weighed, measured and identified the sex of a total of 137 nestlings from 43 nests (Table 1). Owl nestlings were measured at age from 1 to 42 days after hatching (n = 418 measurements). Each nestling was measured and weighted on average 3.1-times (SE = 0.1, n = 137 nestlings) during its stay on the nest. The body mass and wing length of the nestlings were measured usually at intervals between one to two weeks. The nestlings were mostly identified according to their rings. When they were too small to be ringed, they were identified according to colour marks under wings or on legs. Number of measurements was during early phases of ontogeny lower compared to late phases (mean chick age during measurement was 21.4 days, SE = 0.4). This was a consequence of tactful research approach during early phase of nestling ontogeny when female often exhibit disturbed behaviour, especially during cold days. Blood samples for molecular sexing (see below) were taken from each nestling by brachial vein puncture under the wing, around 14–21 days after hatching.

**Table 1:** Prey abundance index (expressed by number of trapped individuals per 100 trap nights) of separate prey groups, number of nests and number of nestlings measured in each study year. Means (per one hectare trapping area or nest) ± SE are shown.

Year	*Apodemus* mice	*Microtus* voles	*Sorex* shrews	Pooled prey abundance	No. nests	No. nestlings per nest
2006	0.00	0.28 ± 0.16	0.00	0.28 ± 0.16	9	3.33 ±0.29
2008	0.64 ± 0.24	0.09 ± 0.09	0.18 ± 0.18	0.92 ± 0.37	6	3.17 ± 0.48
2009	0.28 ± 0.00	0.64 ± 0.24	0.00	0.92 ± 0.24	7	2.00 ± 0.22
2010	3.49 ± 1.30	1.93 ± 0.64	0.09 ± 0.09	5.51 ± 1.88	6	5.83 ± 0.75
2011	0.00	0.18 ± 0.09	0.00	0.18 ± 0.09	5	2.00 ± 0.55
2012	1.56 ± 0.60	0.28 ± 0.16	0.28 ± 0.16	2.11 ± 0.72	2	5.00 ± 0.00
2014	0.28 ± 0.28	0.09 ± 0.28	0.09 ± 0.09	0.46 ± 0.24	8	2.38 ± 0.46

### Prey abundance

The abundance of small mammals was assessed using snap-traps (baited with flour roasted on bacon). Trapping was carried out at the beginning of June each year by setting up snap-traps in three one-hectare areas (11 × 11 trap grid; span of 10 m). The traps were left out for three days and checked every morning. We calculated the abundance index of small mammals as the number of captured individuals per 100 trap nights in each trapping area. All captured mammals (n = 186 individuals, 7 years) were identified to species level and grouped into three prey categories according to the Tengmalm’s owl diet: *Microtus* voles (field vole *M*. *agrestis*, common vole *M*. *arvalis* and European pine vole *M*. *subterraneus*), *Apodemus* mice (yellow-necked mouse *A*. *flavicollis* and wood mouse *A*. *sylvaticus*), and *Sorex* shrews (common shrew *S*. *araneus* and pygmy shrew *S*. *minutus*) (for details see [34,41]).

### Laboratory work

Sex identification from blood samples was carried out using molecular biology techniques involving PCR with primers related to the CHD gene [42]. Genomic DNA from blood samples was extracted by alkaline lysis following a neutralization step [43]. A small amount (3–5 μl) of blood suspension in 96% ethanol was briefly spun down, alcohol was decanted and samples were dried out at room temperature. Into each sample, 50 μl of alkaline lysis buffer (25 mM NaOH, 0.2 mM disodium EDTA, pH 12) was added and heated to 96°C for 10 min. The samples were then cooled down on ice and 50 μl of neutralization buffer (40 mM Tris-HCl, pH 5) was added. As a template for PCR, 1–2 μl of mixture was used. Alternatively, the Chelex100 Resin (BioRad) DNA extraction method can also be used [44]. The 150 μl of 5% Chelex100 solution in sterile water was mixed in a microcentrifuge tube with the blood sample, briefly vortexed and incubated at 56°C for 30 min. After another vortexing followed an 8 min incubation step at 96°C, and samples were then vortexed again. The final step was centrifugation at 10 000 g for 10 min. The supernatant was directly used as a template for PCR. Each PCR reaction was performed in 20 μl. We used the primers 2550F(5'-GTTACTGATTCGTCTACGAGA-3') and 2718R (5'-ATTGAAATGATCCAGTGCTTG-3'), as described by Fridolfsson and Ellegren [42]. The PCR mastermix was prepared according to the Taq DNAPolymerase (NewEngland Biolabs) manufacturer’s guidelines. The PCR conditions were optimized to initial denaturation for 5 min at 95°C, followed by 29 cycles of denaturation at 95°C for 30 s, annealing at 60°C for 40 s, an extension at 72°C for 1 min 10 s, and a final extension at 72°C for 5 min. At the end of the PCR program, the amplified samples were loaded into wells on agarose gel (1% w/v) stained with ethidium bromide. Bands along the DNA molecular marker were detected and photographs taken under UV light. In Tengmalm’s owl, this pair of primers produces a single Z-band (700 bp) in males, and Z- (700 bp) and W- (1200 bp) bands in females [45].

### Statistical analyses

We ran GLMM Gaussian models with an identity link function for testing the effect of (i) year, (ii) brood size, (iii) hatching order and (iv) sex on body mass and wing length (dependent variables) data using R software, version 3.02 [46]. The distribution of dependent variables did not differ from the Gaussian distribution (Kolmogorov-Smirnov tests, *P* at least 0.4). A data unit was represented by each measurement; we used nestling individual (n = 137 individuals; categorical data) and nest (n = 43; categorical data) as random factors, and age of nestling (1–42 days; continuous data) as a covariate. Hatching order (1–2, 3–4, 5–8; categorical data), sex (male/female; categorical data), brood size (1–8; continuous data) and year (n = 7; categorical data) were used as independent (tested) variables. We used factor year in GLMM analysis instead of direct prey abundances to eliminate pseudoreplications and more detailed analyses on our first prediction are described below. We used the following model formula: lmer (dependent variable ~ age of nestling + tested factors + (1| nestling individual) + (1|nest box)). Statistical significance was obtained by comparing each model with previous model using the anova command, starting with comparison of first model with factor with null model without factors. We used forward selection, factors were added to the model based on Akaike’s information criterion (AIC) using Akaike’s weights. Percentages of variability explained by the tested factors were computed as the ratio between decrease of deviance of each model and previous model. We also show the values of the Chi-squared statistic. Because body mass was not meaningful to show standing alone, we present results of above mentioned analysis as body mass increase per day (g/day). Post-hoc comparisons of changes in daily increase of body mass among years and hatching order categories were calculated in R software using glht function.

To support above mentioned models, we also calculated growth and showed parameters for each year, hatching order group and sex using logistic growth curves fitted by non-linear regression based on the formula *y* = *A* / (1 + e ^ (−*K* × (*x* − *T*_*i*_)) (Starck and Ricklefs [47], where *y* is nestling body mass, *x* is nestling age, *A* is an asymptotic—maximal—value, *K* is growth rate, and *T*_*i*_ is the inflexion point). Iterations were carried out using non-linear estimation in Statistica v. 12. 9 [48]. Using this software, we also calculated linear regressions for particular relationships between the above-mentioned parameters of the growth curve and prey abundance (*Microtus* voles, *Apodemus* mice, and pooled prey abundance index, which included *Microtus* voles, *Apodemus* mice and *Sorex* shrews). We present all relevant data used in the analyses in (S1 Dataset).

## Results

The abundance index of the most frequent prey groups of Tengmalm’s owl in our study area varied among years: *Apodemus* mice were the most abundant prey group overall and had the most pronounced between-year fluctuations (mean ± SE; 1.14 ± 1.57 individuals per 100 trap nights), followed by *Microtus* voles (0.48 ± 0.59 individuals per 100 trap nights), and *Sorex* shrews (0.14 ± 0.18 individuals per 100 trap nights) (Table 1).

**Fig 1:**
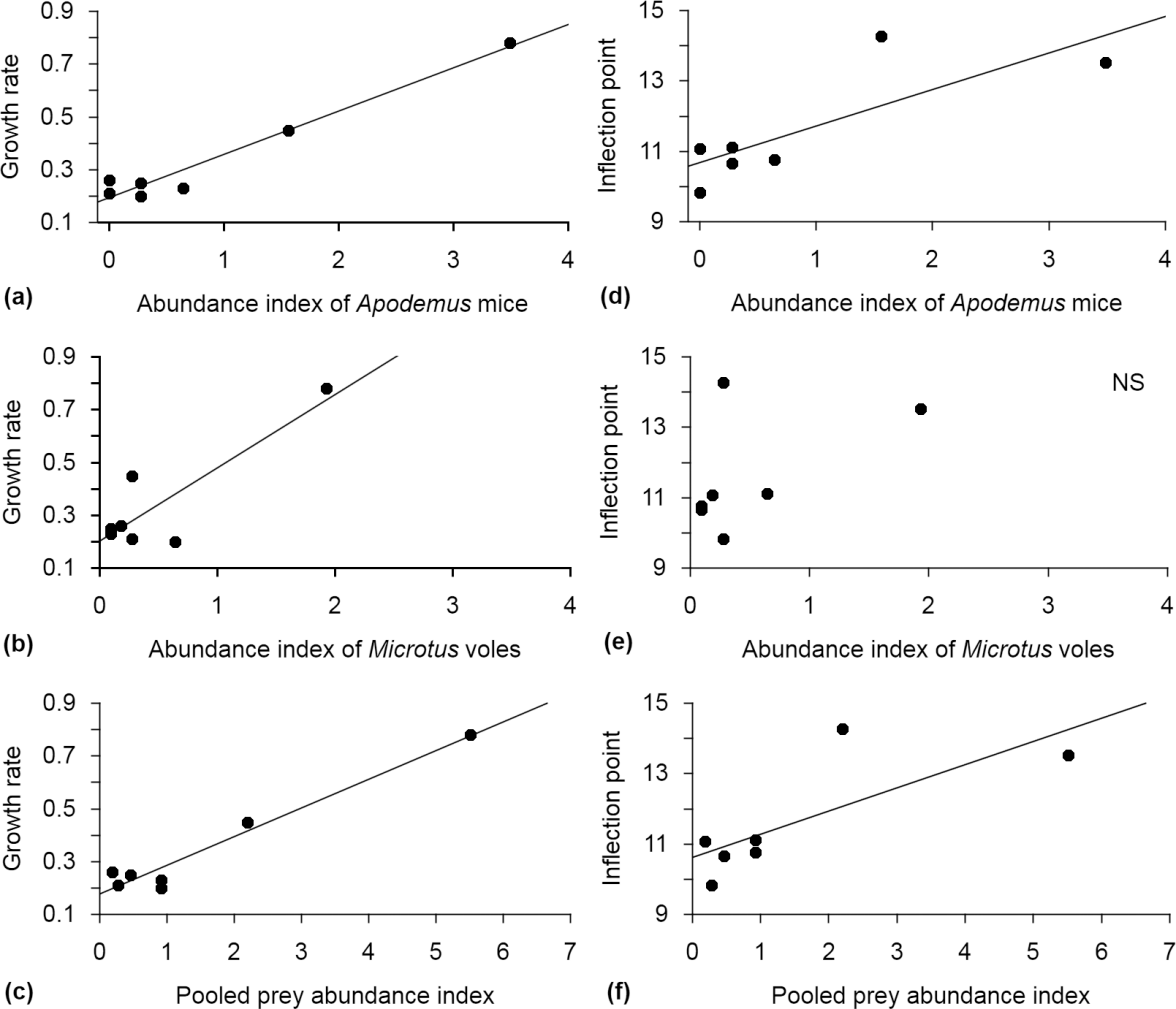
Relationships between the growth rate of Tengmalm’s owl nestlings and prey abundance index (number of individuals per 100 trap nights) of Apodemus mice (a), Microtus voles (b) and index of pooled prey abundance (c), and the relationship between the inflection point of Tengmalm’s owl nestlings and prey abundance index of Apodemus mice (d), Microtus voles (e) and index of pooled prey abundance (f). Lines denote curves fitted by regression.

We found that nestling body mass, controlled for age, significantly varied among study years, hatching order groups and sexes (Table 2). Daily increase of body mass among years varied between 4.4 and 6.5 (mean ± SE, 5.4 ± 0.2, Post-hoc Tukey test, minimal p < 0.01 between maximum and minimum in the years 2008 and 2011, respectively). Within a brood, the older nestlings reached a larger daily body mass increase (min-max: 5.2–5.5, mean ± SE; 5.4 ± 0.1, Post-hoc Tukey test, minimal p = 0.07 between first and last age categories). Body mass increase was higher in females compared to males (5.7 ± 0.1 and 5.2 ± 0.1, respectively). These results can be also supported by comparing growth parameters between above mentioned categories; early hatched young showed higher asymptote and inflexion point, and lower growth rate than late hatched young (Table 3). Similarly, females reached higher asymptote and inflection point, and lower growth rate than males (Table 3). After hatching (1–3 d after hatching), females were on average 2.1 g (n = 11 individuals) heavier than males, and they were also 8.2 g (n = 52 individuals) heavier during the fledging period (i.e., 30–36 d after hatching; Fig 1). Brood size was not recommended to be added to the model using the AIC criterion. Similarly, no factor was recommended to be added to the model when nestling wing length was a dependent variable.

**Table 2:** Effects of the tested factors on nestling body mass, based on Gaussian GLMM models with nestling age as covariate and nestling individual and nest as random factors (n = 137 nestlings from 43 broods). Explained variability is shown in cumulative way.

Model	Explained variability (%)	DF	Chi	p
Mass ~ Year	0.6	236	24.1	< 0.01
Mass ~ Year + Sex	0.7	235	7.3	< 0.01
Mass ~ Year + Sex + Hatching order group	0.9	233	8.3	0.02

**Table 3:** Parameters of the growth curve for Tengmalm’s owl nestlings related to year, hatching order and the sex of nestlings. The asymptote (maximal body mass), inflection point and growth rate are shown (n = number of nestlings measured; n_m_ = number of measurements).

	Asymptote (g)	Inflection point (days)	Growth rate	n	n_m_
*Hatching order*					
1–2	131.7	10.1	0.24	80	229
3–4	126.9	9.6	0.23	41	137
5–8	123.6	9.4	0.28	16	52
*Sex*					
Female	134.6	10.1	0.22	62	195
Male	124.8	9.6	0.27	75	223
*Year*					
2006	135.0	9.8	0.21	30	100
2008	150.4	10.8	0.23	19	26
2009	142.7	11.1	0.20	14	42
2010	125.8	13.5	0.78	35	116
2011	115.4	11.1	0.26	10	28
2012	123.7	14.3	0.45	10	32
2014	130.1	10. 7	0.25	19	74

**Fig 2:**
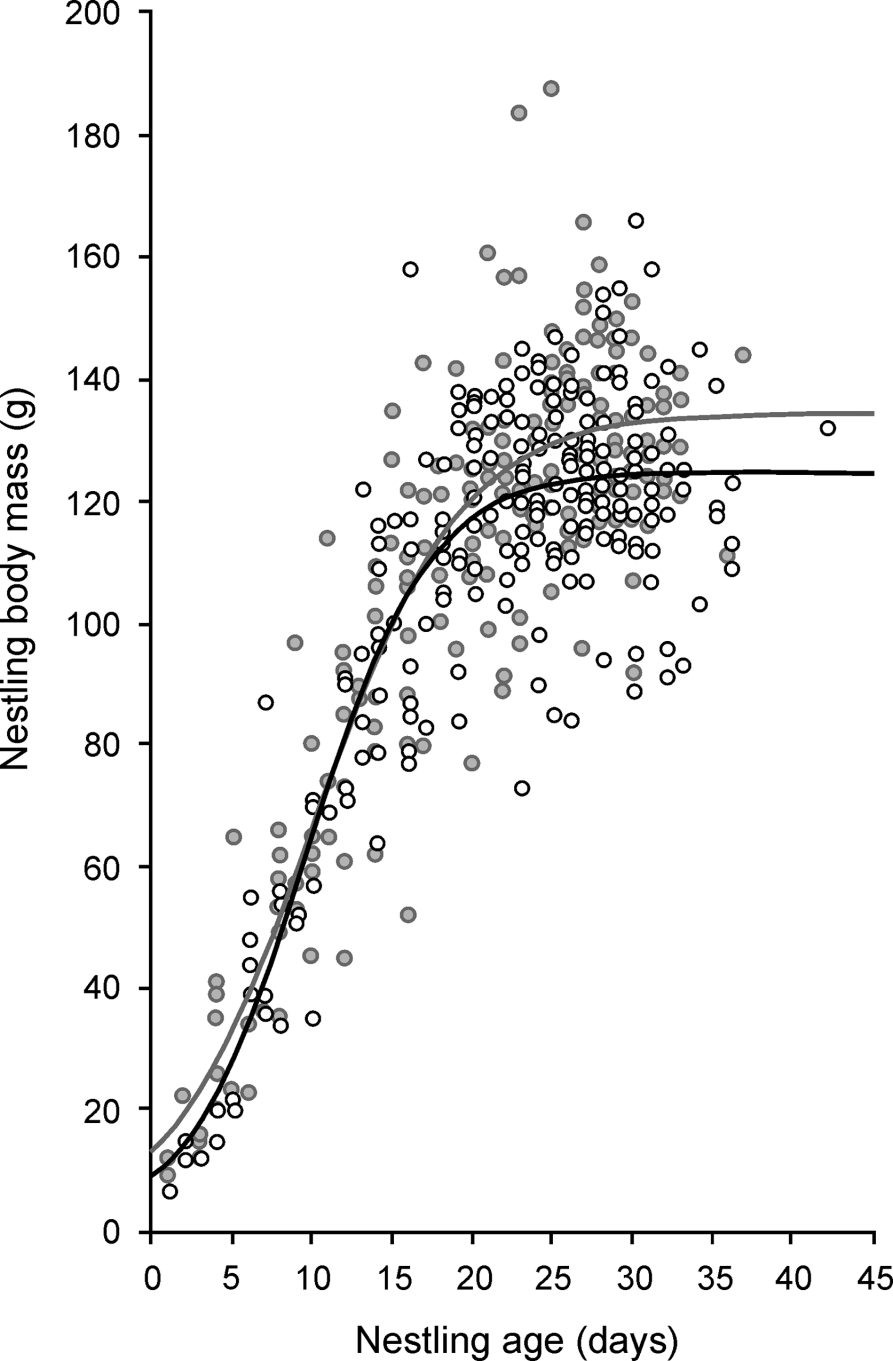
Logistic growth curve for body mass of male (n = 75 individuals) and female (n = 62 individuals) Tengmalm’s owl nestlings. Open circles and black line represent males; filled circles and grey line represent females. Formula for females: *y* = 134.62 / (1 + e^ (−0.22 × (*x* − 10.19))); formula for males: *y* = 124.80 / (1 + e^ (−0.27 × (*x* − 9.61))).

Further analyses on growth parameters showed that growth rate was positively correlated with *Apodemus* mouse index (regressions, n = 7 years: beta = 0.979, *F* = 114.25, p < 0.01; Fig 2A), *Microtus* vole index (beta = 0.862, *F* = 14.464, p = 0.01; Fig 2B) and pooled prey abundance index (beta = 0.980, *F* = 119.94, p < 0.01; Fig 2C). Inflection point was positively correlated with *Apodemus* mouse index (beta = 0.799, *F* = 8.818, p = 0.03; Fig 2D) and pooled prey abundance index (beta = 0.864, *F* = 14.764, p = 0.01; Fig 2F). No relationship was found between inflection point and *Microtus* vole index (beta = 0.521, *F* = 1.862, p = 0.23; Fig 2E) or *Sorex* shrew index (beta = 0.645, *F* = 3.556, p = 0.12), nor growth rate and *Sorex* shrew index (beta = 0.314, *F* = 0.546, p = 0.50). Finally, the yearly asymptote of body mass was not related to the abundance of any prey group index (*Apodemus* mice, beta = −0.22, *F* = 0.244, p = 0.64; *Microtus* voles, beta = −0.16, *F* = 0.14, p = 0.73; *Sorex* shrews, beta = 0.058, *F* = 0.017, p = 0.90; pooled prey abundance index, beta = −0.30, *F* = 0.486, p = 0.52).

## Discussion

### Body mass

In accordance with our first hypothesis, we found that the body mass of nestlings controlled for age varied among years. Simultaneously, the yearly growth rate and inflection point increased (i.e., nestlings grew faster and for a longer period) with increasing prey abundance index. This effect was most pronounced during the peak years of *Apodemus* mice and *Microtus* voles. No relationship was found between the asymptote of nestling body growth (i.e. maximal nestling mass) and prey abundance index. Eldegard & Sonerud [10] documented that supplemental feeding led to parental allocation of food for self-maintenance rather than to higher body mass of Tengmalm’s owl nestlings. On the other hand, Hipkiss et al. [3] showed that the asymptote of Tengmalm’s owl nestling body mass increased in experimentally fed broods, but no relationship was found between the rate of mass gain and food supplementation. In this light, Kouba et al. [49] demonstrated that Tengmalm’s owl parents produced nestlings with different body mass during a two-year period with extremely different prey abundance (*Apodemus* mouse peak compared to a poor rodent year), but Valkama et al. [16] found that the body condition (expressed by residuals of the regression between body mass and wing length) of fledged Tengmalm’s owlets was not associated with the phase of the vole cycle. Similarly inconsistent results were documented in other owl species, e.g. supplementary food resources have been found to affect the growth rate of little owl *Athene noctua*, burrowing owl *Athene cunicularia* or Ural owl *Strix uralensis* nestlings [4,12,19], however, the body condition of Montagu’s harrier *Circus pygargus* during fledging (expressed by the difference between measured body mass and the asymptotic body mass) did not vary according to vole cycles [30]. We suggest that the body mass of the Tengmalm’s owl nestlings in a temperate areas varies under natural prey abundance, but other factors, such as clutch size adjustment by females [50,51], the feeding effort of males [52,53], or variable environmental conditions (e.g. the night length which can limit foraging time of owls, as well as the level of interspecific competition with other vole-eating predators [50,54]), may play important roles.

The body mass of nestlings can be affected by brood size. For example, Eurasian kestrel *Falco tinnunculus* nestlings showed increased mortality and reduced growth rate when broods were experimentally enlarged, even when parents increased their hunting effort [22]. Similarly, experimental brood enlargement in barn owl *Tyto alba* led to increased nestling mortality and decreased body mass of the surviving male and female nestlings [24]. Also, in a northern population of Tengmalm’s owl, nestlings in larger broods suffered from higher mortality and fledglings from larger broods tended to reach a lower mass than those in reduced or control broods [16,55]. We found no effect of brood size on nestling body mass in the natural environments of this temperate Tengmalm’s owl population, thus rejecting our second hypothesis. Tengmalm’s owl females in temperate areas adjusted their clutch size better than females in northern areas, where prey availability is less predictable, which can lead to a clutch size larger than optimal and increased nestling mortality [50]. In light of the mentioned effect of latitude, we suggest that no effect of brood size on nestling body mass was found in our study population because females adjusted their clutch and brood size depending on prey availability.

In agreement with our third hypothesis, we found that early-hatched nestlings reached a larger body mass, and showed higher asymptote and inflection point, compared to late-hatched nestlings. Similarly, in Montagu’s harrier, Arroyo et al. [30] found the poorest fledgling condition in the youngest nestlings. Also, in other owl species (e.g., little owl, burrowing owl, Ural owl), hatching order has been found to affect growth rate [4,12,19]. In our study, late-hatched nestlings suffered from poor body condition, supporting that hatching asynchrony in Tengmalm’s owl is an adaptive way to optimize brood size under varying environmental conditions (see also [16]).

In keeping with our fourth hypothesis, we found that female nestlings reached a higher body mass than male nestlings during their time in the nest, and thus a higher asymptote and inflexion point was found in females compared to males. This finding corresponds with the results of Hipkiss et al. [3], who suggested that the female’s larger size gives them a competitive advantage over their male siblings during fights over food items [56].

### Wing length

Despite adult female Tengmalm’s owls having slightly longer wings than males, no male-female difference in wing length was found in nestlings. Similarly, in marsh harrier *Circus aeruginosus*, Krijgsveld et al. [28] found no difference between the sexes in wing length, although female fledglings were heavier than males. Our results also agree with the finding of Hipkiss et al. [3] that juvenile Tengmalm’s owl females require more time to fully develop their wings than males. Furthermore, we found no effect of year, brood size, hatching order or prey abundance index on the wing length of nestlings. These findings are in contrast to the two-year study performed in the same study area by Kouba et al. [49], in which owl parents produced nestlings with longer wings during an *Apodemus* peak year (2010), compared to a poor rodent year (2011). In our seven-year dataset (2006–2014, excluding 2007 and 2013), we recorded no occurrence of extremely high prey abundance, except in one year (2010). The disunity of these results indicates that wing length can differ under extremely different food conditions, but the differences are not pronounced when food availability is relatively stable. This finding is quite unexpected, because the individual duration of nestling periods is related to wing length rather than body mass, sex, prey abundance index or weather conditions [5].

## Conclusions

No factor affected wing length growth, while the body mass of nestlings controlled for age varied among years, hatching order groups and sexes. Growth parameters were affected by prey abundance and also differed between sexes and with hatching order. Thus, the body mass of nestlings seems to be more sensitive than wing length to environmental factors. The latter is probably controlled more by the physiology of the species.

## Supporting Information

S1 Dataset Relevant data used in the analyses. (PDF)
