# Supplementary material for: Factors Affecting Growth of Tengmalm’s Owl (Aegolius funereus) Nestlings: Prey Abundance, Sex and Hatching Order
Source: PLoS One. 2015 Oct 7;10(10):e0138177. doi: 10.1371/journal.pone.0138177 (PMC4596578; doi:10.1371/journal.pone.0138177)
Supplement: S1 Dataset — (PDF) [file pone.0138177.s001.pdf]

box	year	mass	sex	hatched group	brood size	age
574	2006	20	M	1	3	5
624	2006	20	M	1	3	4
554	2006	61	F	1	4	12
409	2006	62	F	1	4	10
504	2006	66	F	1	5	8
624	2006	71	M	1	3	10
20	2006	73	F	1	3	12
92	2006	73	M	1	2	12
504	2006	97	F	1	5	9
91	2006	98	F	1	6	16
409	2006	106	F	1	4	16
541	2006	111	M	1	3	26
624	2006	113	M	1	3	30
91	2006	114	F	1	6	22
20	2006	121	F	1	3	17
554	2006	125	F	1	4	20
541	2006	126	M	1	3	20
624	2006	127	M	1	3	17
92	2006	132	M	1	2	19
504	2006	132	F	1	5	21
504	2006	135	F	1	5	15
574	2006	138	M	1	3	26
92	2006	139	M	1	2	25
92	2006	140	M	1	2	31
624	2006	141	M	1	3	23
554	2006	145	F	1	4	26
504	2006	147	F	1	5	29
20	2006	153	F	1	3	30
20	2006	157	F	1	3	23
574	2006	12	F	1	3	3
624	2006	15	M	1	3	2
92	2006	35	M	1	2	10
409	2006	49	F	1	4	8
504	2006	55	M	1	5	6
624	2006	56	M	1	3	8
554	2006	57	M	1	4	10
409	2006	62	F	1	4	14
20	2006	65	F	1	3	10
92	2006	83	M	1	2	17
504	2006	87	M	1	5	7
91	2006	96	M	1	6	14
541	2006	98	M	1	3	24
91	2006	105	M	1	6	20
20	2006	113	F	1	3	15
541	2006	115	M	1	3	18
554	2006	117	M	1	4	18
554	2006	117	M	1	4	31
574	2006	117	F	1	3	30
624	2006	117	M	1	3	15
504	2006	122	M	1	5	13
624	2006	123	M	1	3	28
92	2006	133	M	1	2	23
574	2006	133	F	1	3	24
504	2006	135	M	1	5	19
504	2006	137	M	1	5	27
624	2006	137	M	1	3	21

92	2006	141	M	1	2	29
554	2006	143	M	1	4	24
20	2006	159	F	1	3	28
20	2006	161	F	1	3	21
624	2006	7	M	2	3	1
574	2006	9	F	2	3	1
20	2006	34	M	2	3	8
624	2006	39	M	2	3	7
504	2006	41	F	2	5	4
409	2006	44	M	2	4	6
554	2006	54	M	2	4	8
504	2006	65	F	2	5	5
409	2006	71	M	2	4	12
20	2006	78	M	2	3	13
91	2006	91	M	2	6	12
554	2006	93	M	2	4	16
541	2006	103	M	2	3	22
91	2006	105	M	2	6	18
554	2006	107	M	2	4	22
624	2006	109	M	2	3	14
20	2006	111	M	2	3	19
541	2006	112	M	2	3	16
504	2006	114	F	2	5	11
554	2006	122	M	2	4	29
624	2006	122	M	2	3	27
574	2006	127	F	2	3	28
574	2006	130	F	2	3	22
624	2006	137	M	2	3	20
20	2006	139	M	2	3	26
504	2006	143	F	2	5	17
504	2006	143	F	2	5	25
504	2006	16	F	2	5	3
554	2006	34	F	2	4	6
504	2006	39	F	2	5	4
504	2006	52	F	2	5	16
91	2006	71	M	2	6	10
91	2006	77	M	2	6	16
554	2006	77	F	2	4	20
554	2006	79	F	2	4	14
504	2006	80	F	2	5	10
554	2006	96	F	2	4	27
504	2006	9	F	3	5	1
504	2006	22	F	3	5	2
504	2006	58	F	3	5	8
650	2008	53	F	1	5	9
577	2008	100	M	1	3	17
405	2008	109	M	1	2	20
402	2008	126	M	1	3	18
650	2008	126	F	1	5	19
79	2008	166	M	1	4	30
66	2008	188	F	1	2	25
650	2008	53	F	1	5	8
577	2008	100	M	1	3	15
405	2008	121	F	1	2	18
650	2008	126	F	1	5	18
402	2008	133	M	1	3	27
79	2008	154	M	1	4	28
402	2008	158	M	1	3	16

66	2008	184	F	1	2	23
650	2008	39	M	2	5	6
577	2008	84	M	2	3	13
402	2008	111	M	2	3	25
402	2008	113	M	2	3	14
650	2008	117	M	2	5	16
79	2008	127	M	2	4	26
650	2008	35	F	2	5	4
650	2008	109	F	2	5	14
79	2008	118	M	2	4	24
650	2008	15	F	3	5	3
650	2008	89	F	3	5	13
849	2009	48	M	1	3	6
850	2009	57	F	1	4	9
20	2009	79	M	1	4	16
542	2009	83	F	1	4	14
626	2009	88	F	1	3	14
67	2009	105	F	1	4	25
406	2009	106	F	1	3	14
849	2009	116	M	1	3	14
850	2009	122	F	1	4	16
850	2009	123	F	1	4	24
406	2009	126	F	1	3	21
849	2009	126	M	1	3	26
849	2009	138	M	1	3	19
542	2009	143	F	1	4	22
67	2009	144	F	1	4	37
542	2009	147	F	1	4	27
406	2009	155	F	1	3	27
626	2009	157	F	1	3	22
20	2009	158	M	1	4	31
626	2009	166	F	1	3	27
849	2009	26	F	1	3	4
850	2009	36	F	1	4	7
542	2009	45	F	1	4	12
20	2009	64	M	1	4	14
849	2009	92	F	1	3	12
406	2009	95	F	1	3	12
406	2009	96	F	1	3	19
850	2009	101	F	1	4	14
67	2009	110	M	1	4	23
542	2009	110	F	1	4	20
849	2009	112	F	1	3	17
849	2009	116	F	1	3	24
542	2009	119	F	1	4	25
406	2009	122	F	1	3	32
406	2009	125	F	1	3	25
850	2009	127	F	1	4	22
542	2009	136	F	1	4	32
67	2009	139	M	1	4	35
20	2009	155	M	1	4	29
850	2009	23	F	2	4	5
850	2009	85	F	2	4	12
850	2009	113	F	2	4	20
901	2010	118	M	1	6	32
620	2010	119	M	1	8	35
901	2010	120	M	1	6	27
620	2010	122	M	1	8	28

620	2010	122	M	1	8	33
901	2010	125	M	1	6	30
620	2010	128	M	1	8	31
504	2010	130	F	1	5	24
905	2010	131	M	1	8	32
905	2010	135	M	1	8	30
504	2010	141	F	1	5	29
905	2010	141	M	1	8	28
307	2010	145	M	1	5	34
307	2010	147	M	1	5	29
307	2010	151	M	1	5	28
502	2010	152	F	1	8	27
620	2010	113	F	1	8	26
620	2010	116	F	1	8	31
620	2010	117	F	1	8	29
901	2010	118	M	1	6	28
901	2010	118	M	1	6	35
504	2010	120	M	1	5	22
905	2010	120	M	1	8	31
620	2010	121	F	1	8	33
502	2010	122	F	1	8	29
504	2010	122	M	1	5	27
901	2010	123	M	1	6	25
905	2010	125	M	1	8	28
905	2010	127	M	1	8	30
901	2010	130	M	1	6	30
905	2010	130	M	1	8	26
502	2010	139	F	1	8	25
307	2010	141	F	1	5	26
901	2010	73	M	2	6	23
901	2010	84	M	2	6	26
901	2010	93	M	2	6	33
901	2010	94	M	2	6	28
901	2010	113	M	2	6	36
620	2010	118	M	2	8	24
905	2010	118	M	2	8	30
905	2010	120	M	2	8	28
502	2010	121	M	2	8	27
905	2010	121	M	2	8	26
620	2010	122	M	2	8	31
620	2010	124	M	2	8	29
620	2010	125	M	2	8	33
504	2010	126	M	2	5	28
502	2010	129	M	2	8	23
620	2010	129	M	2	8	27
504	2010	131	M	2	5	20
905	2010	131	M	2	8	24
901	2010	132	M	2	6	42
307	2010	138	F	2	5	32
307	2010	140	F	2	5	26
504	2010	147	M	2	5	25
307	2010	153	F	2	5	30
620	2010	111	F	2	8	36
504	2010	112	M	2	5	30
504	2010	113	M	2	5	18
504	2010	114	M	2	5	28
504	2010	116	M	2	5	26
905	2010	125	M	2	8	28

504	2010	126	M	2	5	23
620	2010	126	F	2	8	22
905	2010	127	M	2	8	26
905	2010	131	M	2	8	24
502	2010	133	M	2	8	21
502	2010	134	M	2	8	25
620	2010	136	F	2	8	31
620	2010	137	F	2	8	33
905	2010	137	M	2	8	22
620	2010	139	F	2	8	25
620	2010	139	F	2	8	27
620	2010	145	F	2	8	29
307	2010	147	F	2	5	30
307	2010	148	F	2	5	25
307	2010	149	F	2	5	28
620	2010	107	M	3	8	31
504	2010	111	F	3	5	16
504	2010	118	F	3	5	26
504	2010	119	F	3	5	28
905	2010	119	M	3	8	29
504	2010	126	F	3	5	21
502	2010	128	F	3	8	27
905	2010	128	M	3	8	26
502	2010	130	F	3	8	29
307	2010	133	M	3	5	28
504	2010	133	F	3	5	24
905	2010	134	M	3	8	22
502	2010	136	F	3	8	26
905	2010	136	M	3	8	20
307	2010	139	M	3	5	26
905	2010	142	M	3	8	24
307	2010	145	M	3	5	23
620	2010	91	M	3	8	32
502	2010	113	M	3	8	29
502	2010	116	M	3	8	27
620	2010	118	M	3	8	29
502	2010	120	M	3	8	24
905	2010	128	M	3	8	27
502	2010	130	M	3	8	25
905	2010	139	M	3	8	24
620	2010	95	M	3	8	30
620	2010	107	M	3	8	27
502	2010	112	M	3	8	22
502	2010	112	M	3	8	25
502	2010	113	M	3	8	30
502	2010	115	M	3	8	27
502	2010	120	M	3	8	23
905	2010	133	F	3	8	22
905	2010	139	F	3	8	25
502	2010	119	F	3	8	23
502	2010	122	F	3	8	20
502	2010	124	F	3	8	21
502	2010	127	F	3	8	28
502	2010	138	F	3	8	25
616	2011	91	F	1	3	22
19	2011	99	F	1	3	21
406	2011	105	M	1	5	20
616	2011	107	F	1	3	30

44	2011	108	F	1	3	21
406	2011	109	M	1	5	36
44	2011	115	F	1	3	27
306	2011	121	M	1	3	20
406	2011	122	M	1	5	29
406	2011	124	M	1	5	23
44	2011	125	F	1	3	30
306	2011	125	M	1	3	32
19	2011	127	F	1	3	28
19	2011	128	F	1	3	31
44	2011	85	M	1	3	25
44	2011	92	M	1	3	19
406	2011	103	M	1	5	34
406	2011	104	M	1	5	18
306	2011	108	F	1	3	18
406	2011	125	M	1	5	27
306	2011	134	F	1	3	30
406	2011	85	M	2	5	16
406	2011	96	M	2	5	32
406	2011	110	M	2	5	25
406	2011	79	M	2	5	14
406	2011	89	M	2	5	30
406	2011	115	M	2	5	23
406	2011	123	M	2	5	36
623	2012	112	M	1	6	31
623	2012	114	M	1	6	24
409	2012	117	M	1	5	31
409	2012	120	M	1	5	24
409	2012	122	F	1	5	22
623	2012	124	F	1	6	22
409	2012	129	F	1	5	32
623	2012	129	F	1	6	29
409	2012	133	F	1	5	29
409	2012	108	F	2	5	20
409	2012	114	F	2	5	27
623	2012	121	F	2	6	20
409	2012	122	F	2	5	30
623	2012	128	F	2	6	30
409	2012	129	F	2	5	33
623	2012	138	F	2	6	27
623	2012	100	F	2	6	18
409	2012	112	F	2	5	18
623	2012	122	F	2	6	28
623	2012	123	F	2	6	25
623	2012	124	F	2	6	31
409	2012	130	F	2	5	31
409	2012	133	F	2	5	25
409	2012	136	F	2	5	28
409	2012	80	F	3	5	16
623	2012	87	M	3	6	16
623	2012	107	M	3	6	26
623	2012	112	M	3	6	23
623	2012	114	M	3	6	29
409	2012	115	F	3	5	26
409	2012	122	F	3	5	23
409	2012	124	F	3	5	29
1330	2014	12	F	1	1	1
1330	2014	20	F	1	1	4

13111	2014	23	F	1	4	6
63	2014	45	F	1	2	10
1348	2014	57	M	1	3	10
13154	2014	62	F	1	4	8
1396	2014	65	F	1	4	11
848	2014	73	M	1	1	12
1330	2014	74	F	1	1	11
63	2014	88	F	1	2	16
848	2014	88	M	1	1	14
13111	2014	88	F	1	4	13
13111	2014	88	F	1	4	16
1377	2014	92	F	1	3	30
1396	2014	101	F	1	4	23
1330	2014	107	F	1	1	16
1348	2014	110	M	1	3	19
63	2014	115	F	1	2	21
1396	2014	117	F	1	4	29
63	2014	118	F	1	2	24
848	2014	118	M	1	1	21
1348	2014	119	M	1	3	25
63	2014	124	F	1	2	32
13154	2014	127	F	1	4	15
848	2014	128	M	1	1	28
1377	2014	130	F	1	3	22
1377	2014	132	F	1	3	20
13154	2014	134	F	1	4	28
1348	2014	136	M	1	3	30
13154	2014	136	F	1	4	25
1330	2014	144	F	1	1	31
13111	2014	147	F	1	4	28
13111	2014	157	F	1	4	23
13111	2014	15	M	1	4	4
13154	2014	36	M	1	4	7
1348	2014	51	M	1	3	9
1396	2014	59	F	1	4	10
13111	2014	69	M	1	4	11
1396	2014	89	F	1	4	22
1377	2014	95	M	1	3	13
13154	2014	98	M	1	4	14
1377	2014	103	M	1	3	22
1348	2014	111	M	1	3	18
1377	2014	116	M	1	3	20
1396	2014	117	F	1	4	28
1348	2014	119	M	1	3	24
1377	2014	125	M	1	3	30
13111	2014	127	M	1	4	21
13154	2014	129	M	1	4	24
13154	2014	130	M	1	4	27
1348	2014	140	M	1	3	29
13111	2014	144	M	1	4	26
13111	2014	12	M	2	4	2
13154	2014	22	M	2	4	5
1348	2014	35	F	2	3	8
13111	2014	52	M	2	4	9
1348	2014	80	F	2	3	17
13111	2014	84	M	2	4	19
13111	2014	90	M	2	4	24
13154	2014	90	M	2	4	12

1348	2014	97	F	2	3	23
1377	2014	124	F	2	3	21
13154	2014	137	M	2	4	25
13154	2014	139	M	2	4	22
1348	2014	141	F	2	3	28
1348	2014	141	F	2	3	33
1377	2014	142	F	2	3	19
13111	2014	142	M	2	4	32
1377	2014	150	F	2	3	29
13154	2014	12	M	2	4	3
13111	2014	36	M	2	4	7
13154	2014	70	M	2	4	10
13154	2014	125	M	2	4	23
13154	2014	129	M	2	4	20

box	year	wing	sex	hatched order	brood size	age
574	2006	17	M	1	3	5
574	2006	106	M	1	3	26
574	2006	15	F	2	3	3
574	2006	98	F	2	3	24
574	2006	120	F	2	3	30
409	2006	29	F	2	4	8
409	2006	48	F	2	4	14
409	2006	30	F	1	4	10
409	2006	55	F	1	4	16
409	2006	21	M	3	4	6
409	2006	42	M	3	4	12
541	2006	83	M	2	3	18
541	2006	109	M	2	3	24
541	2006	89	M	1	3	20
541	2006	115	M	1	3	26
541	2006	69	M	3	3	16
541	2006	96	M	3	3	22
91	2006	50	M	4	6	10
91	2006	71	M	4	6	16
91	2006	65	F	1	6	16
91	2006	97	F	1	6	22
91	2006	63	M	2	6	14
91	2006	90	M	2	6	20
91	2006	59	M	3	6	12
91	2006	90	M	3	6	18
574	2006	11	F	3	3	1
574	2006	88	F	3	3	22
574	2006	106	F	3	3	28
504	2006	25	M	2	5	6
504	2006	40	M	2	5	7
504	2006	60	M	2	5	13
504	2006	91	M	2	5	19
504	2006	118	M	2	5	27
504	2006	30	F	1	5	8
504	2006	45	F	1	5	9
504	2006	68	F	1	5	15
504	2006	96	F	1	5	21
504	2006	123	F	1	5	29
92	2006	31	M	1	2	12
92	2006	63	M	1	2	19

92	2006	85	M	1	2	25
92	2006	115	M	1	2	31
20	2006	28	F	2	3	10
20	2006	80	F	2	3	21
20	2006	86	F	2	3	15
20	2006	115	F	2	3	28
20	2006	35	F	1	3	12
20	2006	52	F	1	3	17
20	2006	86	F	1	3	23
20	2006	120	F	1	3	30
504	2006	20	F	3	5	4
504	2006	32	F	3	5	5
504	2006	50	F	3	5	11
504	2006	82	F	3	5	17
504	2006	115	F	3	5	25
504	2006	14	F	4	5	3
504	2006	19	F	4	5	4
504	2006	36	F	4	5	10
504	2006	55	F	4	5	16
504	2006	11	F	5	5	1
504	2006	15	F	5	5	2
504	2006	27	F	5	5	8
92	2006	20	M	2	2	10
92	2006	40	M	2	2	17
92	2006	69	M	2	2	23
92	2006	97	M	2	2	29
20	2006	19	M	3	3	8
20	2006	32	M	3	3	13
20	2006	61	M	3	3	19
20	2006	94	M	3	3	26
554	2006	30	M	2	4	10
554	2006	71	M	2	4	18
554	2006	94	M	2	4	24
554	2006	125	M	2	4	31
554	2006	32	F	1	4	12
554	2006	75	F	1	4	20
554	2006	96	F	1	4	26
624	2006	15	M	1	3	4
624	2006	34	M	1	3	10
624	2006	72	M	1	3	17
624	2006	115	M	1	3	23
624	2006	128	M	1	3	30
624	2006	14	M	2	3	2
624	2006	29	M	2	3	8
624	2006	72	M	2	3	15
624	2006	93	M	2	3	21
624	2006	128	M	2	3	28
554	2006	25	M	3	4	8
554	2006	51	M	3	4	16
554	2006	76	M	3	4	22
554	2006	106	M	3	4	29
554	2006	19	F	4	4	6
554	2006	56	F	4	4	14
554	2006	75	F	4	4	20
554	2006	102	F	4	4	27
624	2006	12	M	3	3	1
624	2006	21	M	3	3	7
624	2006	55	M	3	3	14

624	2006	88	M	3	3	20
624	2006	117	M	3	3	27
405	2008	104	M	1	2	20
405	2008	102	F	2	2	18
66	2008	122	F	2	2	23
66	2008	127	F	1	2	25
402	2008	63	M	3	3	14
402	2008	107	M	3	3	25
650	2008	28	F	1	5	9
650	2008	75	F	1	5	19
650	2008	27	F	2	5	8
650	2008	73	F	2	5	18
577	2008	53	M	2	3	15
577	2008	58	M	1	3	17
577	2008	49	M	3	3	13
650	2008	20	M	3	5	6
650	2008	68	M	3	5	16
650	2008	17	F	4	5	4
650	2008	56	F	4	5	14
650	2008	15	F	5	5	3
650	2008	48	F	5	5	13
79	2008	94	M	1	4	30
79	2008	93	M	2	4	28
79	2008	91	M	3	4	26
79	2008	72	M	4	4	24
402	2008	96	M	1	3	18
402	2008	92	M	2	3	16
402	2008	128	M	2	3	27
849	2009	29	F	2	3	4
849	2009	60	F	2	3	12
849	2009	80	F	2	3	17
849	2009	102	F	2	3	24
67	2009	86	F	1	4	25
67	2009	116	F	1	4	37
406	2009	65	F	1	3	14
406	2009	96	F	1	3	21
406	2009	114	F	1	3	27
406	2009	50	F	2	3	12
406	2009	80	F	2	3	19
406	2009	99	F	2	3	25
406	2009	119	F	2	3	32
542	2009	25	F	2	4	12
542	2009	62	F	2	4	20
542	2009	86	F	2	4	25
542	2009	115	F	2	4	32
626	2009	60	F	1	3	14
626	2009	100	F	1	3	22
626	2009	113	F	1	3	27
849	2009	39	M	1	3	6
849	2009	88	M	1	3	14
849	2009	103	M	1	3	19
849	2009	130	M	1	3	26
850	2009	18	F	3	4	5
850	2009	38	F	3	4	12
850	2009	78	F	3	4	20
850	2009	19	F	2	4	7
850	2009	48	F	2	4	14
850	2009	95	F	2	4	22

850	2009	26	F	1	4	9
850	2009	57	F	1	4	16
850	2009	100	F	1	4	24
20	2009	48	M	1	4	16
20	2009	116	M	1	4	31
20	2009	35	M	2	4	14
20	2009	104	M	2	4	29
67	2009	84	M	2	4	23
67	2009	120	M	2	4	35
542	2009	48	F	1	4	14
542	2009	83	F	1	4	22
542	2009	108	F	1	4	27
502	2010	121	F	1	8	27
502	2010	118	F	2	8	25
502	2010	110	M	3	8	23
502	2010	102	M	4	8	21
504	2010	106	F	1	5	24
504	2010	106	M	2	5	22
504	2010	84	M	3	5	20
504	2010	77	M	4	5	18
504	2010	123	M	4	5	30
620	2010	102	M	1	8	28
620	2010	92	M	3	8	24
620	2010	98	F	2	8	26
620	2010	85	F	4	8	22
620	2010	130	F	4	8	33
620	2010	137	F	4	8	36
504	2010	64	F	5	5	16
504	2010	90	F	5	5	21
504	2010	121	F	5	5	28
901	2010	97	M	2	6	25
901	2010	132	M	2	6	35
901	2010	110	M	1	6	27
905	2010	90	M	5	8	20
905	2010	114	M	5	8	26
905	2010	124	M	5	8	29
905	2010	97	M	4	8	22
905	2010	120	M	4	8	28
905	2010	100	M	3	8	24
905	2010	124	M	3	8	30
905	2010	104	M	2	8	26
905	2010	128	M	2	8	31
905	2010	108	M	1	8	28
307	2010	121	M	1	5	28
307	2010	142	M	1	5	34
307	2010	116	F	2	5	26
307	2010	105	F	3	5	26
307	2010	133	F	3	5	32
502	2010	122	F	5	8	26
502	2010	106	M	6	8	24
502	2010	124	M	6	8	29
502	2010	94	M	7	8	22
502	2010	118	M	7	8	27
502	2010	127	M	7	8	30
502	2010	85	F	8	8	20
502	2010	108	F	8	8	25
502	2010	120	F	8	8	28
307	2010	101	F	4	5	25

307	2010	128	F	4	5	30
307	2010	95	M	5	5	23
307	2010	120	M	5	5	28
905	2010	109	M	6	8	24
905	2010	115	M	6	8	27
905	2010	93	F	7	8	22
905	2010	114	F	7	8	25
620	2010	123	M	5	8	31
620	2010	108	M	6	8	29
620	2010	116	M	6	8	32
620	2010	88	M	7	8	27
620	2010	94	M	7	8	30
901	2010	43	M	3	6	23
901	2010	80	M	3	6	33
901	2010	91	M	3	6	36
901	2010	112	M	3	6	42
406	2011	60	M	2	5	18
406	2011	106	M	2	5	27
406	2011	122	M	2	5	34
406	2011	46	M	4	5	14
406	2011	85	M	4	5	23
406	2011	106	M	4	5	30
406	2011	124	M	4	5	36
406	2011	66	M	1	5	20
406	2011	80	M	1	5	23
406	2011	105	M	1	5	29
406	2011	124	M	1	5	36
406	2011	50	M	3	5	16
406	2011	90	M	3	5	25
406	2011	115	M	3	5	32
306	2011	84	M	1	3	20
306	2011	125	M	1	3	32
306	2011	83	F	2	3	18
306	2011	130	F	2	3	30
616	2011	78	F	1	3	22
616	2011	110	F	1	3	30
19	2011	80	F	1	3	21
19	2011	113	F	1	3	28
19	2011	120	F	1	3	31
44	2011	96	F	1	3	21
44	2011	122	F	1	3	27
44	2011	130	F	1	3	30
44	2011	76	M	2	3	19
44	2011	103	M	2	3	25
409	2012	90	M	1	5	24
409	2012	118	M	1	5	31
409	2012	84	F	3	5	20
409	2012	108	F	3	5	27
409	2012	118	F	3	5	30
409	2012	130	F	3	5	33
409	2012	81	F	2	5	22
409	2012	110	F	2	5	29
409	2012	120	F	2	5	32
623	2012	86	M	1	6	24
623	2012	118	M	1	6	31
623	2012	84	F	2	6	22
623	2012	112	F	2	6	29
623	2012	81	F	3	6	20

623	2012	112	F	3	6	27
623	2012	122	F	3	6	30
623	2012	66	F	4	6	18
623	2012	102	F	4	6	25
623	2012	116	F	4	6	28
623	2012	127	F	4	6	31
409	2012	62	F	4	5	18
409	2012	100	F	4	5	25
409	2012	114	F	4	5	28
409	2012	125	F	4	5	31
409	2012	48	F	5	5	16
409	2012	82	F	5	5	23
409	2012	95	F	5	5	26
409	2012	106	F	5	5	29
623	2012	53	M	5	6	16
623	2012	85	M	5	6	23
623	2012	100	M	5	6	26
623	2012	110	M	5	6	29
1396	2014	26	F	2	4	10
1396	2014	74	F	2	4	22
1396	2014	97	F	2	4	28
1396	2014	131	F	2	4	34
1348	2014	22	M	2	3	9
1348	2014	70	M	2	3	18
1348	2014	91	M	2	3	24
1348	2014	111	M	2	3	29
1348	2014	27	M	1	3	10
1348	2014	72	M	1	3	19
1348	2014	94	M	1	3	25
1348	2014	115	M	1	3	30
1396	2014	30	F	1	4	11
1396	2014	82	F	1	4	23
1396	2014	101	F	1	4	29
1396	2014	131	F	1	4	35
1348	2014	20	F	3	3	8
1348	2014	58	F	3	3	17
1348	2014	64	F	3	3	23
1348	2014	92	F	3	3	28
1348	2014	93	F	3	3	33
63	2014	32	F	1	2	10
63	2014	42	F	1	2	16
63	2014	56	F	1	2	21
63	2014	61	F	1	2	24
63	2014	109	F	1	2	32
1377	2014	44	M	2	3	13
1377	2014	83	M	2	3	20
1377	2014	95	M	2	3	22
1377	2014	127	M	2	3	30
848	2014	36	M	1	1	12
848	2014	46	M	1	1	14
848	2014	85	M	1	1	21
848	2014	113	M	1	1	28
1377	2014	87	F	1	3	20
1377	2014	95	F	1	3	22
1377	2014	112	F	1	3	30
1377	2014	73	F	3	3	19
1377	2014	87	F	3	3	21
1377	2014	122	F	3	3	29

13154	2014	24	F	1	4	8
13154	2014	59	F	1	4	15
13154	2014	78	F	1	4	18
13154	2014	117	F	1	4	25
13154	2014	123	F	1	4	28
13154	2014	17	M	2	4	7
13154	2014	52	M	2	4	14
13154	2014	67	M	2	4	17
13154	2014	116	M	2	4	24
13154	2014	120	M	2	4	27
13154	2014	15	M	3	4	5
13154	2014	39	M	3	4	12
13154	2014	52	M	3	4	15
13154	2014	92	M	3	4	22
13154	2014	118	M	3	4	25
13154	2014	11	M	4	4	3
13154	2014	30	M	4	4	10
13154	2014	43	M	4	4	13
13154	2014	85	M	4	4	20
13154	2014	104	M	4	4	23
13111	2014	14	F	1	4	6
13111	2014	40	F	1	4	13
13111	2014	53	F	1	4	16
13111	2014	97	F	1	4	23
13111	2014	115	F	1	4	28
13111	2014	12	M	2	4	4
13111	2014	32	M	2	4	11
13111	2014	82	M	2	4	21
13111	2014	109	M	2	4	26
13111	2014	10	M	3	4	2
13111	2014	25	M	3	4	9
13111	2014	56	M	3	4	19
13111	2014	78	M	3	4	24
13111	2014	92	M	3	4	32
1330	2014	10	F	1	1	1
1330	2014	13	F	1	1	4
1330	2014	35	F	1	1	11
1330	2014	59	F	1	1	16
1330	2014	125	F	1	1	31
13111	2014	19	M	4	4	7
